# Antibiotic Exposure in a Low-Income Country: Screening Urine Samples for Presence of Antibiotics and Antibiotic Resistance in Coagulase Negative Staphylococcal Contaminants

**DOI:** 10.1371/journal.pone.0113055

**Published:** 2014-12-02

**Authors:** Anne Mette Lerbech, Japheth A. Opintan, Samuel Oppong Bekoe, Mary-Anne Ahiabu, Britt Pinkowski Tersbøl, Martin Hansen, Kennedy T. C. Brightson, Samuel Ametepeh, Niels Frimodt-Møller, Bjarne Styrishave

**Affiliations:** 1 Toxicology Laboratory, Analytical Biosciences, Department of Pharmacy, Faculty of Health and Medical Sciences, University of Copenhagen, Copenhagen, Denmark; 2 Department of Microbiology, University of Ghana Medical School, P. O. Box KB 4236, Accra, Ghana; 3 Department of International Health, Immunology and Microbiology, Faculty of Health Sciences, University of Copenhagen, Copenhagen, Denmark; 4 Maternal Health Unit, Shai-Osudoku District Hospital, Dodowa, Ghana; 5 Microbiology Laboratory, Shai-Osudoku District Hospital, Dodowa, Ghana; 6 Department of Clinical Microbiology, Hvidovre Hospital, Kettegaards alle 30, 2650, Hvidovre, Copenhagen, Denmark; University Medical Center Utrecht, Netherlands

## Abstract

Development of antimicrobial resistance has been assigned to excess and misuse of antimicrobial agents. Staphylococci are part of the normal flora but are also potential pathogens that have become essentially resistant to many known antibiotics. Resistances in coagulase negative staphylococci (CoNS) are suggested to evolve due to positive selective pressure following antibiotic treatment. This study investigated the presence of the nine most commonly used antimicrobial agents in human urine from outpatients in two hospitals in Ghana in relation to CoNS resistance. Urine and CoNS were sampled (n = 246 and n = 96 respectively) from patients in two hospitals in Ghana. CoNS were identified using Gram staining, coagulase test, and MALDI-TOF/MS, and the antimicrobial susceptibility to 12 commonly used antimicrobials was determined by disk diffusion. Moreover an analytical method was developed for the determination of the nine most commonly used antimicrobial agents in Ghana by using solid-phase extraction in combination with HPLC-MS/MS using electron spray ionization. The highest frequency of resistance to CoNS was observed for penicillin V (98%), trimethoprim (67%), and tetracycline (63%). *S. haemolyticus* was the most common isolate (75%), followed by *S. epidermidis* (13%) and *S. hominis* (6%). *S. haemolyticus* was also the species displaying the highest resistance prevalence (82%). 69% of the isolated CoNS were multiple drug resistant (≧4 antibiotics) and 45% of the CoNS were methicillin resistant. Antimicrobial agents were detected in 64% of the analysed urine samples (n = 121) where the most frequently detected antimicrobials were ciprofloxacin (30%), trimethoprim (27%), and metronidazole (17%). The major findings of this study was that the prevalence of detected antimicrobials in urine was more frequent than the use reported by the patients and the prevalence of resistant *S. haemolyticus* was more frequent than other resistant CoNS species when antimicrobial agents were detected in the urine.

## Introduction

The development of effective antimicrobial agents has been accompanied by the emergence of drug resistant organisms, and has arisen as one of the most serious health care problems in the world, especially in low and middle income countries [1].

Development of antimicrobial resistance has been assigned to excess and misuse of antimicrobial agents which provide selective pressure favouring the appearance of resistant strains [2]. In developing countries such as Ghana, increasing prevalence of resistance towards several antimicrobial groups has been reported in various bacterial pathogens [3]–[5]. A study performed on Gram-negative bacteria isolated from hospitalized patients showed high prevalences of resistance to tetracycline (82%), ampicillin (75%), chloramphenicol (75%), and co-trimoxazole (72%) [3]. In addition, more than 80% of *Escherichia coli* strains that were isolated from stools of healthy volunteers in Ghana exhibited resistance to ampicillin, tetracycline, chloramphenicol and co-trimoxazole [6]. These studies do not only indicate a widespread prevalence of antimicrobial resistance in the most important bacterial pathogens in Ghana [6], but also that resistance is a serious problem both for community-acquired as well as for nosocomial infections (hospital-acquired).

In Ghana, antibiotics are widely available to the public, from a variety of sources, including hospitals and pharmacies, licensed medicine stalls and drugstores, roadside stalls and peddlers [7], [8]. Despite prohibitive legislations, antibiotics can be purchased without prescription [9]–[11]. This widespread availability has led to inappropriate use by patients and healthcare providers [8], [10], [12]. Furthermore, antimicrobial therapy is mainly empirical due to the relative lack of appropriate laboratory facilities for culture and susceptibility testing of bacteria [3], [13]. The extensive use of antibiotics and the absence of susceptibility testing have led to a steady increase in antimicrobial drug resistance.

Coagulase-negative staphylococci (CoNS) are frequently distributed on the human skin and mucuous membranes and have long been regarded as harmless skin commensals and dismissed as culture contaminants, for example in urine cultures [14]. It is well known that cutaneous antibiotic resistant CoNS are subject to positive selective pressure following antibiotic treatment [15]. The anatomical site of the selective antibiotic pressure has not yet been identified. However, antibiotics given orally or parenterally, must reach the skin surface, to be able to interfere with the skin flora [16], [17]. Since antibiotics are distributed in body fluids as well as tissues, it is likely that antibiotics are excreted to the skin, e.g. by sweat glands [16], [17], and thereby enhancing the selective antibiotic pressure on the cutaneous flora, making the skin surface a possible significant pool of antibiotic resistance genes [15], [16]. CoNS from the skin also commonly contaminate urine samples. In this context, antimicrobial resistance to CoNS may be a good general indicator for antibiotic drug exposure although it cannot replace a proper surveillance system that measure the exact quantities of antibiotics sold.

Humans may also be exposed to antibiotics indirectly, i.e. through food, water and/or herbal medicines. Antimicrobial agents are also used in livestock to treat, prevent, and control diseases and to enhance feed efficiency and weight gain [18]. As in human medicine, the use of antimicrobial agents in veterinary medicine creates a selective pressure for the emergence and dissemination of antimicrobial-resistant bacteria, including animal pathogens, human pathogens that have food animal reservoirs, and commensal bacteria that are present in livestock [2], [19], [20]. It is well documented that these resistant bacteria can be transferred to humans either by direct contact with animals or through the food [21]. Also, herbal medicines are intensively used in Ghana [22] but it is presently unknown to what extent such products contain antibiotics.

The present study investigated the occurrence of antimicrobial resistance in CoNS and the presence of 9 commonly used antimicrobial agents [23]–[25] in human urine from outpatients in two hospitals in Ghana. Furthermore, we investigated if there was a relation between the incidence of resistant CoNS and the presence of antibiotics in urine.

## Materials and Methods

### Ethics statement

Ethical clearance for this study was obtained from the Committee on Human Research Publication and Ethics, School of Medical Science, Komfo Anokye Teaching Hospital. Only adults participated in the study, and all participants provided informed consent in writing.

### Sampling

246 urine samples were collected from 246 adult outpatients (males and females aged 18 to 85 years) at two hospitals in Ghana, Korle-Bu Teaching Hospital (KBTH) and Shai Osudoku District Hospital (SODH), between February and April 2012. All adult patients who reported to the medical laboratories in KBTH and SODH with a request for urine, blood, or stool analysis were included in the study. Patients unable to speak English, Akan, Ga, or Ewe were excluded from the study.

To obtain information on demographics, medical history, and antibiotic consumption, qualitative interview was conducted with each patient by trained anthropologists. In the interviews, patients were asked if they had taken any antibiotics within the past 14 days. During the interviews a catalogue of different antibiotic formulations and brands were presented to patients in case of difficulties in remembering name(s) of drug(s) consumed. Interviews were conducted in English or in the local languages of Akan, Ga, and Ewe, and lasted between 7 and 25 minutes. The interviews were recorded and subsequently transcribed into English. The study was thoroughly explained to patients and consent requested. All patients approached agreed to participate in the study.

After the interview, patients were handed a sterile plastic container and asked to provide about 150 mL of mid-stream urine sample. Urine sample from each patient was divided into two aliquots; 2 mL for microbial analysis and the remaining for pharmaceutical analysis. 100 µL internal standard (d3-trimethoprim (11.56 µg/mL), d8-ciprofloxacin (12.03 µg/mL), d5-penicillin (1.02 µg/mL) (Qmx Laboratories, England), and d4-sulfamethoxazole (10.00 µg/mL)) (Toronto Research Chemicals, Canada) was added to the aliquots for pharmaceutical analysis, and frozen at −18°C for further processing.

### Culture and susceptibility testing

10 µL fresh urine was inoculated on a Discovery agar plate (Statens Serum Institut, Hillerød, Denmark), and incubated for 18–24 h at 35°C in ambient air. Colony morphologies indicative of Staphylococcus were sub-cultured onto Mueller-Hinton agar (Becton-Dickinson, USA) and incubated for 24–48 h at 35°C in ambient air. Coagulase negative Staphylococci (CoNS) were identified by Gram staining and coagulase tube test. Further confirmation and speciation of CoNS was done at Hvidovre Hospital, Denmark, using Matrix-Assisted Laser Desorption-Ionization Time-of-Flight Mass Spectrometry (MALDI-TOF/MS) (Bruker Biotype system (Microflex LT/SH MS)). A score value greater than 1.8 was accepted as identified, using the FlexiControl and Biotyper real-time classification (RTC) software. *Staphylococcus aureus* ATCC 25923 and *Escherichia coli* ATCC 25922 were used as control strains. *Staphylococcus saprophyticus* was excluded from this study, due to its known ability to cause UTI in females compared to other CoNS which rarely causes infections.

Antimicrobial susceptibility to penicillin, cefoxitin, vancomycin, erythromycin, chloramphenicol, clindamycin, gentamicin, tetracycline, fusidic acid, rifampicin, sulfonamide, and trimethoprim was determined by disk diffusion test or the E-test on Mueller-Hinton agar (Becton-Dickinson, USA) in accordance with guidelines from the European Committee on Antimicrobial Susceptibility Testing (EUCAST) [26]. Antibiotic discs were purchased from Oxoid (Denmark) or bioMérieux SA (France), and *Escherichia coli* ATCC 25922 was used as reference strain. CoNS isolates simultaneously resistant to ≧4 antibiotics were defined as multiple drug resistant (MDR).

### Analysis of antibiotics in urine

#### Reagents and standards

Amoxicillin was purchased from Duchefa (Holland). Ampicillin, metronidazole, cefuroxime, ciprofloxacin, trimethoprim, and sulfamethoxazole were purchased from Fluka (Denmark). Tetracycline hydrochloride was purchased from Sigma-Aldrich, and doxycycline hydrochloride from Nycomed (Denmark). The internal standard (IS) d4-sulfamethoxazole was purchased from Toronto Research Chemicals Canada), and the ISs d3-trimethoprim and d8-ciprofloxacin from Qmx Laboratories (England). Acetonitrille and methanol were of HPLC grade and purchased from Lab-Scan (Poland). Ammonium formate (≧99.0%), formic acid (98–199%), and triethylamine (≧99%) were purchased from Merck KGaA (Germany), and Sigma-Aldrich (Denmark), respectively. Pure water was produced in house with a Milli-Q water gradient system (Millipore, Bedford, MA, USA).

A standard antibiotic-mix with a concentration of 10 µg/mL was prepared by mixing a known amount of each stock solution with methanol in a 10 mL volumetric flask. Likewise an internal standard solution with a concentration 10 µg/mL for each IS was prepared. This standard antibiotic-mix was used to prepare all standard solutions used in all analysis. The standard antibiotic-mix was protected from light and stored at −18°C.

#### Solid phase extraction

Sample extraction was performed on Oasis HLB 6 cm^3^ 200 mg (30 µm) cartridges from Waters (Milford, MA, USA). Cartridges were conditioned with 2 mL MeOH followed by 2 mL 0.01 M citrate buffer and 2 mL distilled water. Thawed urine samples were weighted and 100 µL citrate buffer was added. The pH of this suspension was adjusted to 7±0.3 with 2 M H_2_SO_4_ or 2 M NaCl, and loaded onto SPE columns with a flow of approximately 1.5 mL/min. After extraction, sorbent was carefully dried under vacuum for up to 1 hour.

Two different solvents, A and B, for eluting antibiotics from the SPE columns were prepared. Solvent A was prepared by mixing 900 mL milliQ-water with 0.35 g ammonium formate followed by 6 mL acetonitrile, 4 mL methanol, 100 µL formic acid, and 100 µL triethylamine. Solvent B was prepared by mixing 5 mL milliQ-water with 0.35 g ammonium formate followed by 650 mL acetonitrile, 30 mL methanol, 100 µL formic acid, and 100 µL triethylamine. Antibiotic extracts were eluted using a mixture of 5 mL Solvent A: Solvent B (10∶90, v/v) and 3 mL methanol: water (80∶20, v/v). The extract was evaporated to dry under a gentle steam of nitrogen at 40°C and re-dissolved in 200 µL mobile phase A: mobile phase B (80∶20, v/v), as described below. Prior to final high-performance liquid chromatography-tandem mass spectrometry (HPLC-MS/MS) analysis the samples were transferred to a 0.2 mL flat cap PCR tube and centrifuged at 10,000 rpm in 3 min.

#### HPLC-MS/MS analysis

All samples were analysed with a HPLC-MS/MS system. The liquid chromatographic system consisted of a 1100 Series HPLC instrument (Agilent Technologies Inc., Palo Alto, CA, USA) equipped with a degasser, a cooled autosampler and a column oven. The mass spectrometer coupled to the HPLC system was a PE-Sciex API 3000 triple quadruple mass spectrometer from Applied Biosystems MDS Sciex Instruments (Foster City, CA, USA) equipped with electrospray ionization (ESI) interface.

The injection volume was set at 10 µL. Separation was performed on a XTerra MS C18 column (100 mm×2.1 mm×3.5 µm) and a guard column XTerra MS RP18 column (2.0 mm×3.0 mm×3.5 µm) both from Waters (Milford, MA, USA) applying a binary gradient flow rate of 250 µL/min at 30°C. Mobile phase A was prepared by mixing 990 mL milliQ-water with 0.35 g ammonium formate followed by 10 mL acetonitrile, 100 µL formic acid, and 100 µL triethylamine. Mobile phase B was prepared by mixing 50 mL milliQ-water with 0.35 g ammonium formate followed by 950 mL acetonitrile, 100 µL formic acid, and 100 µL triethylamine. The initial mobile phase composition was 99% A and 1% B and was kept for 5 min followed by a 15 min gradient ending with a composition of 25% A and 75% B. This composition was held for 1 min thereafter returning to the starting conditions giving a total analysis time of 20 min. Retention times and HPLC-MS/MS settings for the individual antibiotics can be found in Table S1.

Electrospray ionization was performed in positive mode (ESI+) in segment 2 and 4 and negative mode (ESI-) in segment 1, 3, and 4. In both cases the temperature was set to 400°C with a nebulizer gas flow of 10 L/min, while the curtain gas flow was 6 mL/min, the collision gas flow was 10 mL/min and ionspray voltage was 3500 and −3500 for positive and negative ionization mode, respectively. The multiple reaction monitoring (MRM) function was applied in all analyses. A chromatogram of the HPLC-MS/MS method is shown in Figure S1.

The linearity, limit of detection (LOD_Instr_) and limit of quantification (LOQ_Instr_) of the HPLC-MS/MS system was evaluated by injecting the standard antibiotic mixture into the HPLC-MS/MS system. Linearity was investigated using a 6-point calibration curve determined in the range of 100-5000 ng/mL. The LOD_Instr_ and LOQ_Instr_ were determined from the standard deviation of the response from the lowest calibration standard (1 µg/mL) injected 6 times and by the slope of the calibration curve.

Fisher's exact test was used to compare frequencies whenever data were separated into two categories e.g. the two categories resistance (resistance/no resistance) and antibiotics (present/not present in the urine). A *p* value <0.05 (two-sided) was considered to be statistical significant. The data were analysed using GraphPad Prism software version 6.0.

## Results

### Participants

246 patients participated in the study. 82% (n = 202) were females of which 44% (n = 88) were pregnant. Patient's age ranged between 15 and 85 years with an average age of 33 years for females and 43 years for males. 79% (n = 193) of the patients had a valid National Health Insurance (NHIS) and 88% (n = 217) had an education (Primary, Secondary or Tertiary).

### Occurrence of CoNS resistance

**Figure 1:**
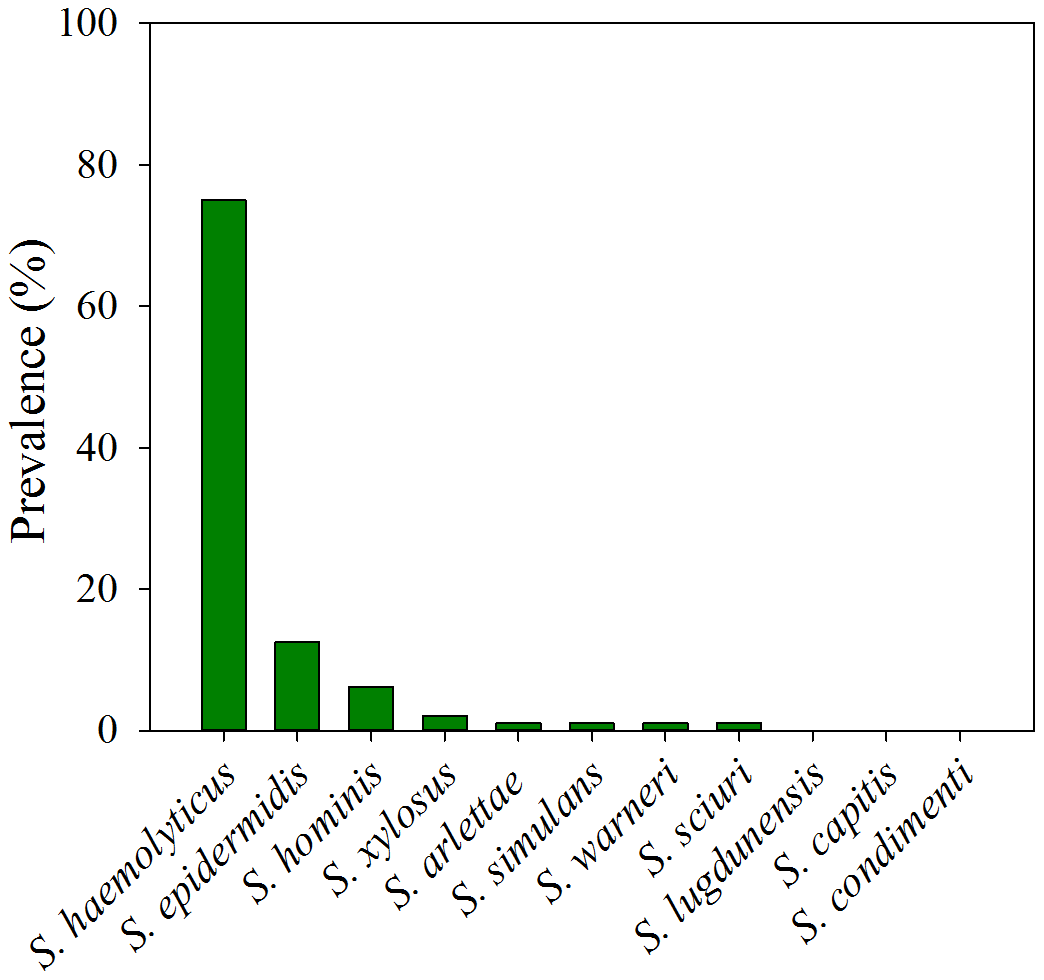
Prevalence of staphylococci isolated from urine samples (n = 96).

In total, 96 CoNS were isolated from 85 patients. The 96 isolates were identified with 54 from KBTH and 42 from SODH. 8 different species of CoNS were identified among the 96 isolates. The most common isolated species was *S. haemolyticus* (75%) followed by *S. epidermidis* (13%) and *S. hominis* (13%) (Figure 1).

**Figure 2:**
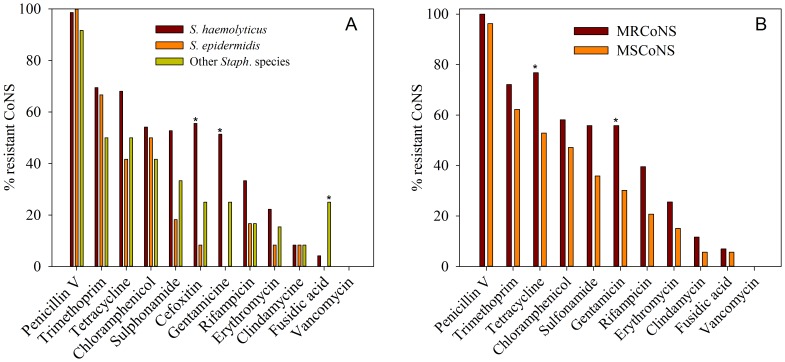
Prevalence of resistant CoNS isolated from urine samples (n = 96). A: Percentage resistant *S. haemolyticus* (red bars, n = 72), *S. epidermidis* (orange bars, n = 12), and other staphylococcal species (green bares, n = 12) to each tested antibiotics. B: Percentage methicillin-resistant CoNS (MRCoNS; red bars, n = 43) and methicillin-susceptible CoNS (MSCoNS; orange bars, n = 53).

Figure 2A shows the fraction of resistant *S. haemolyticus*, *S. epidermidis* and other isolated staphylococcal species to each tested antimicrobial. 98% of the CoNS isolates were resistant to penicillin V followed by 67% resistance to trimethoprim and 63% to tetracycline. *S. haemolyticus* (red bars) and other isolated staphylococcal species (green bars) exhibited resistance to all antibiotics except vancomycin. *S. epidermidis* (orange bars) showed resistance to all antibiotics except gentamicin and vancomycin. *S. haemolyticus* showed a higher frequency of resistance to gentamicin and cefoxitin compared to *S. epidermidis* (Fisher's exact test, *p* = 0.008 and *p* = 0.036, respectively), and a lower frequency of resistance to fusidic acid compared to the other staphylococcal species (*p*<0.036). For the remaining antimicrobials, no statistical significant difference was observed among the CoNS species. In total, 69% (66/96) of the CoNS were MDR, with *S. haemolyticus*, *S. epidermidis* and the other CoNS being 78% (56/72), 33% (4/12) and 50% (6/12) MDR, respectively.

Figure 2B shows the antibiotic resistance in methicillin-resistant CoNS (MRCoNS) and methicillin-susceptible CoNS (MSCoNS). Of the 96 isolated CoNS, 45% (43/96) were resistant to cefoxitin, and were considered as methicillin resistant. Methicillin resistance was predominantly observed in *S. haemolyticus* (91% (39/43)), followed by other staphylococcal species (7.0% (3/43)), and *S. epidermidis* (2.3% (1/43)). MDR were likewise more frequently observed in *S. haemolyticus* (63% (33/53)), compared to *S. epidermidis* (21% (11/53)), and other staphylococcal species (17% (9/53)). MRCoNS displayed a significantly higher frequency of resistance to tetracycline and gentamicin than MSCoNS (*p* = 0.0194 and *p* = 0.0134, respectively).

**Figure 3:**
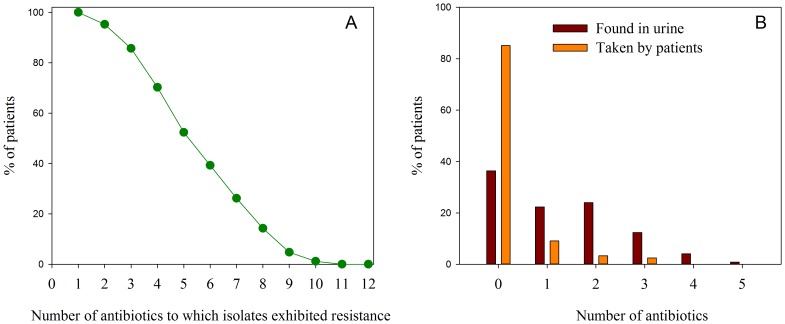
Antibiotics in patients. A: Percentage of patients relative to the number of antibiotics to which isolates exhibited resistance (n = 84). B: Percentage of patients (n = 121) relative to the number of antimicrobial agents found in patient's urine (red bars, n = 155) and the number of antibiotic claimed to be taken by patients (orange bars, n = 25).

The antimicrobial susceptibility was tested against 12 different antibiotic markers. No resistance was found against vancomycin and it was consequently not included in the following assessments. Figure 3A shows the number of resistant antibiotic markers for the urine samples collected versus the percentage of patients. A single *S. haemolyticus* isolate (corresponding to 1.2%) was resistant to 10 antibiotic markers, 52% (44/84) of the isolates were resistant to 5 antibiotic markers, and 100% (84/84) of the patients were resistant to at least 1 antibiotic marker.

### Presence of antimicrobial agents in urine samples

The prevalence of antimicrobial agents in the urine samples was determined by SPE-HPLC-MS/MS. In total, 121 urine samples (83 patients with CoNS isolates and 38 with no CoNS) were analysed by HPLC-MS/MS. Table 1 indicates the number of each antimicrobial detected in the urine separated into samples in which CoNS where isolated (n = 112) or where no CoNS were isolated (n = 43). 9 different antimicrobial agents were detected in urine from patients. The most frequently detected antimicrobial agent was ciprofloxacin (30%, (46/155)) followed by trimethoprim (27%, (41/155)), metronidazole (17%, (28/155)), and sulfamethoxazole (13%, (20/155)). Of the 83 analysed urine samples from patients with isolated CoNS, 55 samples contained antimicrobial agent. For the 38 patients analysed with no isolated CoNS, 22 patients had antimicrobial agents in their urine. Comparing patients with CoNS isolated in urine and those with no CoNS isolated, there was no significant difference in the prevalence of antimicrobial agents in the urine samples (*p* = 0.4186). Table 1 also shows the minimum, maximum and median urine concentrations (ng/mL) for each antibiotic. Ciprofloxacin was found in the highest concentration (2.47·10^5^ ng/mL). In contrast, ampicillin was only found in 2 patients and the concentrations were below LOQ.

**Table 1:** Occurrence of antimicrobial agents in urine collected from patients from KBTH in Accra and SODH in Dodowa.

Antimicrobial agent	Occurrence of antimicrobial agents				Urine concentration (ng/mL)		
	Isolated CoNS		Non-isolated CoNS		min	max	median
Amoxicillin	3		5		<LOQ	7.66•10^2^	5.15
Metronidazole	22		4		<LOQ	3.86•10^4^	<LOQ
Ampicillin	2		0		<LOQ	<LOQ	-
Tetracycline	1		1		3.41•10^1^	9.54•10^2^	-
Doxycycline	3		0		4.89•10^2^	2.21•10^3^	8.14•10^2^
Cefuroxime	4		3		<LOQ	7.33•10^1^	1.01
Sulfamethoxazole	12		8		<LOQ	5.01•10^3^	1.85
Ciprofloxacin	35		11		<LOQ	2.47•10^5^	3.33
Trimethoprim	30		11		<LOQ	4.05•10^3^	<LOQ

In total, the 9 antimicrobials were detected 112 times in patients from whom CoNS were isolated (n = 83) and 43 times in patients from whom no CoNS were isolated (n = 38). Also shown are the minimum, maximum, and median concentrations (ng/mL) of the antimicrobial agents. For patients with isolated CoNS, antimicrobial agents were detected in 55 of the 83 samples. For patients from whom no CoNS were isolated, 22 of the 38 urine samples contained antimicrobial agents. <LOQ: S/N<10.

Figure 3B illustrates the percentage of patients with a given number of antimicrobial agents found in the urine (red bars) and the number of antibiotics taken by patients according to interview responses (orange bars). 86% (104/121) of the patients responded not to have taken any antibiotics but only 36% (44/121) of the samples did not contain antimicrobial agents. Overall, antimicrobial agents were detected in urine from 77 out of 121 patients of which only 16 patients responded to have taken antibiotics (Figure 3B). Of the 44 samples with no antimicrobial agents detected one patient responded to have taken antibiotics. This means that more antimicrobial agents than reported by the patients were measured in the urine samples (*p* = 0.0028). Ciprofloxacin was detected in 46 samples but only 4 patients claimed to have taken ciprofloxacin. Two out of 41 patients had taken trimethoprim, 4 out of 26, metronidazole, and 2 out of 20, sulfamethoxazole.

The combined microbial and chemical results are shown in Table 2. The distribution of the staphylococcal species, their antibiotic-resistance profiles, and the samples from which antimicrobial agents were detected in urine are all shown. For the 83 patients with isolated CoNS, *S. haemolyticus* were isolated from 65 patients of whom 45 patients had antimicrobial agents in their urine. For the 18 patients where other resistant staphylococcal species were isolated 10 patients had antimicrobial agents in their urine. This means that patients with detected antimicrobial agents in their urine were not more frequently colonized with resistant *S. haemolyticus* than with other resistant CoNS species (*p* = 0.3983). However, at the species level there was a statistically significant difference between the number of resistant *S. haemolyticus* and the number of resistant non-*S. haemolyticus* when compared to samples with detected and no detected antimicrobials (*p* = 0.0063) i.e. the prevalence of resistant *S. haemolyticus* was higher than the prevalence of resistant non-*S. haemolyticus* when antimicrobials were detected in urine.

**Table 2:** Summary of the microbial and chemical results. 96 species were isolated from 85 patients of which 83 samples were analysed by LC-MS/MS.

	Antibiotic detected		No antibiotic detected		Total
Isolated CoNS (%)	60	(62.5)	36	(37.5)	96
Distribution of staph. species					
*S. haemolyticus* (%)	47	(65.3)	25	(34.7)	72
*non - S. haemolyticus* (%)	13	(54.2)	11	(45.8)	24
Number of resistant and susceptible species					
*S. haemolyticus* (R) (%)	267	(45.4)*	106	(38.4)*	373
non-*S. haemolyticus* (R) (%)	47	(28.0)	37	(30.8)	84
*S. haemolyticus* (I+S) (%)	321	(54.6)	170	(62.6)	491
non-*S. haemolyticus* (I+S) (%)	121	(72.0)	83	(69.2)	204
Number of patients with resistant species					
*S. haemolyticus* (R) (%)	45	(69.2)	20	(30.8)	65
non-*S. haemolyticus* (R) (%)	10	(55.6)	8	(44.4)	18
					

For patients with isolated CoNS, antibiotics were detected in 55 out of 83 samples. For patients with no CoNS isolates, 22 out of 38 urine samples contained antibiotics. The distribution of staphylococcal species, number of staphylococcal isolates and their antibiotic-resistance (R) and antibiotic-susceptibility (I+S), are shown in relation to the samples where antibiotics were detected and samples where no antibiotics were detected. *: Significant differences in resistance between *S. haemolyticus* and non-*S*. *haemolyticus* (p = 0.0063).

Patient age and gender exerted no influence on the occurrence of resistant CoNS or antibiotics in the urine. Also, no significant difference was observed in the occurrence of resistant CoNS between subjects with health insurance and without health insurance. In fact, the occurrence was identical (*p* = 1.00). Furthermore, no effects of education on CoNS resistance (*p* = 0.542) and urine antibiotic content were observed (p = 0.762).

## Discussion

The frequency of resistance to penicillin, trimethoprim, tetracycline and chloramphenicol was high in the isolated CoNS. High frequency of resistance to these drugs have been reported for a number of organisms for several years [27], [28]. No resistance was found against vancomycin, which is beneficial since vancomycin is considered as last drug of choice for treating staphylococcal infections [29]. The most used antibiotics in developing countries are penicillin, cotrimoxazole and tetracycline, as these drugs are inexpensive, relatively broad-spectrum, and considered essential [30]. In Africa, penicillin has been the drug of choice in treating yaws infections, and tetracycline was the empiric drug of choice for cholera, both for prophylaxis as well as treatment. Tetracycline has subsequently been replaced by trimethoprim-sulfamethoxazole, and more recently, quinolones, due to the emergence and spread of resistant strains [9]. The high occurrence of resistance found in the current study is therefore not surprising considering that misuse or high consumption of antimicrobial drugs provide a selective pressure favouring the appearance of resistant strains.

The antibiotic resistance profile observed in the current study is similar to those reported by a recent study in Ghana [3] showing high prevalence of resistance to tetracycline, chloramphenicol, cotrimoxazole, and penicillin. Another study from 2010 investigated Staphylococcus in faecal samples from children [31] and found resistance to the same antibiotics as tested in the current study, in the similar order, but with lower percent resistant strains.

Antimicrobials are known to influence the human microflora, including CoNS that forms part of the normal human skin flora. This means that antimicrobial resistance in CoNS may be an indicator for antibiotic drug use. Very little has been reported from Ghana on the actual use of antibiotics by humans due to the weak systems for the regulations of medicines. Data on the quantities of antibiotics sold are therefore extremely difficult to obtain. A study by Germer and Sinar [32] quantified the drug consumption in Greater Accra region, where both KBTH and SODH are located. Among the antimicrobial agents tested in the current study for resistance, trimethoprim followed by penicillin V, sulfamethoxazole, erythromycin, tetracycline, clindamycin, and chloramphenicol were reported by Germer and Sinar [32] as the most frequently sold antibiotics. This indicates that the total use of antibiotics may correlate with the detected antimicrobial agents in urine and may be fairly consistent with the resistance observed among the CoNS in this study, since the highest resistance observed was to penicillin, trimethoprim, tetracycline and sulfamethoxazole.

CoNS are major components of the human skin with *S. epidermidis* as the predominant species followed by *S. hominis*, and *S. haemolyticus* [29], [33]–[35]. However, the most frequently isolated species in the current study was *S. haemolyticus* (75%) followed by *S. epidermidis* (13%) and *S. hominis* (6%). Moreover *S. haemolyticus* showed a higher frequency of resistance than the other isolated CoNS, a result also observed by Froggatt et al. [36], Lebeaux et al. [37], Barros et al. [38]. These findings emphasize that the population of susceptible bacterial strains is considerably reduced, increasing the prevalence of resistant organisms that can proliferate due to reduced competition [39], [40].

Multi-drug resistance is common among CoNS [41]–[43] which was confirmed in the present study. A study by Cove et al. [15] found that almost 50% of the CoNS isolated from untreated subjects expressed multi-drug resistance. Moreover, Akinkunmi and Lamikanra [44] found the majority (approximately 90%) of their isolated CoNS to be MDR. Aglald-Öhman et al. [43] also found that 59% of the CoNS isolated from patients from an intensive care unit were resistant to at least four out of seven tested antibiotics. Additionally, multi-drug resistance is more common in *S. haemolyticus* than among the other CoNS [45], [46]. This is in agreement with the findings in the current study, where *S. haemolyticus* showed higher prevalence of multiple resistance (78%) compared to *S. epidermidis* (33%), and the other staphylococcal species (50%). In a recent study by Barros et al. [38], 75% of *S. haemolyticus* were MDR, which is similar to the 78% found in the current study.

The prevalence of MRCoNS carriage in the present study was 45%. Compared to a study on nasal carriage of MRCoNS in adults by Barbier et al. [47] this present study, in general, observed a higher resistance to the tested antimicrobial agents. Fusidic acid is not a registered drug in Ghana, which may explain the relatively low resistance towards this antimicrobial. Likewise a study by Lebeaux et al. [37] and by Akinkunmi and Lamikanra [48] found a lower resistance levels among MRCoNS. There are several reports that suggest the transfer of SCCmec from MRCoNS to methicillin-susceptible *Staphylococcus aureus* (MSSA) resulting in the creation of a new methicillin-resistant *Staphylococcus aureus* (MRSA) clone that could result in a potential outbreak [37], [47], [49], [50]. MRSA infections are becoming difficult to treat because resistance to many present antimicrobial classes are developing [47], [51]. Until the 1990s MRSA was almost limited to hospital and healthcare settings but community-acquired strains have now arisen worldwide and currently account for a high proportion of serious infections [41]. On this basis, it is of great concern that 45% of the isolated species in this current study are MRCoNS especially because the CoNS are common on the human skin from where they may spread from person to person.

In Ghana, self-treatment with allopathic medicines including antibiotics is a common practice [11] and this may possibly explain the high prevalence of antimicrobial agents in urine in the current study. Secondly, KBTH is a referral health facility, and patients may have sought care elsewhere and given antibiotics before reporting. In Ghana, adult literacy levels are low [52], and the inconsistencies in patients' responses to interview questions on antibiotic use and findings from urine analysis could in some cases be due to the inability of patients to identify drugs used as antibiotics despite the aids engaged during interviews. Since the current study was health facility-based, the potential for social desirability bias exist and this may also account for some of the inconsistencies [53]. Another more concerning explanation of the high prevalence of antimicrobial agents in urine samples in the current study may be antibiotic consumption from unknown or diffuse sources, for instance through drinking water, milk, tea, food or herbal medicines. If this is the case, then the presence of antibiotics in urine may not only be a snapshot of exposure related to treatment of infections, but also an indicator for chronic exposure from diffuse sources. K'oreje and colleagues [54] analysed river water in Kenya, and found trimethoprim and sulfamethoxazole present in concentrations up to 5 and 20 µg/L, respectively [54]. In the current study, the highest frequency of antimicrobial resistances in CoNS was observed for penicillin V, trimethoprim, chloramphenicol and sulfonamide. We speculate that both trimethoprim and sulfamethoxazole are present in the environment or in food. Moreover, several drugs are used in livestock in Ghana, especially in poultry, either prophylactically or for treating infections. These include tetracyclines, penicillins, sulfonamides, chloramphenicol, tylosin and streptomycin [55]–[57]. The extent to which degree these antibiotics will be present in the surface water, ground water, drinking water and food is unknown.

CoNS are usually collected using nasal swabs. However, nasal swaps, or even skin swabs, are considered invasive sampling. Urine sampling is non-invasive and can be performed by the subjects themselves without any interference from the health personnel. In developing countries with very high rates of tuberculosis, AIDS and other severe diseases and a general public skepticism towards health personnel, obtaining ethical clearance for invasive sampling when non-invasive alternatives exist, is difficult. Consequently, the present study exploited the fact that antibiotics and CoNS can be analysed in the same urine samples. To the best of our knowledge, no other studies have investigated the connection between the occurrence of resistant *S. haemolyticus* and the presence of low concentrations of antibiotics in urine from humans who have not intentionally consumed antibiotics. The exposure to low concentrations of antibiotics will promote the emergence of resistant bacteria. However, it is surprising that the relative low concentrations of antibiotics detected in the urine samples apparently select for resistance, especially because the antibiotics have to be excreted to the skin to influence the CoNS. If this is possible the findings in the present study is worrisome since genes encoding for resistance in CoNS can be transferred to susceptible CoNS [58] and in worse cases to other bacteria, which can cause serious infections. Further research is needed to confirm the relationship between antibiotics in urine and the development of resistance in CoNS strains. Furthermore, it should be stressed that the presence of antibiotics in urine of members of the general public is unknown and that the present findings may not be indicative of exposure in the general population of the country or the city.

## Conclusion

The present study investigated occurrence of resistance in CoNS in human urine along with the occurrence of 9 of the most commonly used antimicrobial agents. It has been shown that the occurrence of drug resistance among CoNS in Ghana was high, and that the CoNS displayed a high prevalence of resistance to methicillin and to multiple antimicrobial agents. In addition, it was established that *S. haemolyticus* was the most distributed species among the isolated CoNS and hence the species exposing the highest antimicrobial resistance.

The major findings in this study were, that:

- the prevalence of detected antimicrobial agents in human urine was more frequent than the use reported by the patients.
- the prevalence of resistant *S. haemolyticus* was more frequent than other resistant coagulase negative staphylococcal species when antimicrobial agents were detected in the urine.

The findings in this study substantiate that antimicrobial resistance among CoNS in Ghana is high compared to resistance observed in industrialized parts of the world. It is worrying that low concentrations of antimicrobial agents were detected in urine from patients who responded that they have not consumed antibiotics, and even more worrisome that these low concentrations may potentially select for antimicrobial resistance. This indicates that there is an urgent need to identify potentially diffuse sources of antibiotic exposure and for more appropriate selection and use of antibiotics in developing countries such as Ghana.

## Supporting Information

Figure S1 **Chromatogram of a standard antibiotic-mix solution in a concentration of 5.0 ppm obtained from the final HPLC-MS/MS method.** The segments are represented in dotted lines.
(PDF)

Table S1 **Compound specific settings in the final analytical LC-MS/MS method, applying ESI in positive and negative mode, together with the most intense precursor ions and product ions for the nine investigated compounds and the three internal standards.** DP: Declustering potential, FP: Focusing potential, EP: Entrance potential, CE: Collision energy, CXP: Collision cell exit potential; V: Volt. Parameters changed from the in-house method are presented in bolt.
(DOC)

Table S2 **Number of staphylococcal isolates and their antibiotic-resistance profiles to individual antimicrobial agents in isolates from KBTH in Accra and SODH in Dodowa.**
(DOC)

Data S1 **Chromatographic and microbiological data for the occurrence of antibiotics and CoNS in human urine from outpatients in two hospitals in Ghana.**
(XLSX)
