# Supplementary figures and images for: Antibiotic Exposure in a Low-Income Country: Screening Urine Samples for Presence of Antibiotics and Antibiotic Resistance in Coagulase Negative Staphylococcal Contaminants
Source: PLoS One. 2014 Dec 2;9(12):e113055. doi: 10.1371/journal.pone.0113055 (PMC4251977; doi:10.1371/journal.pone.0113055)

Supporting Information

Figure S1:

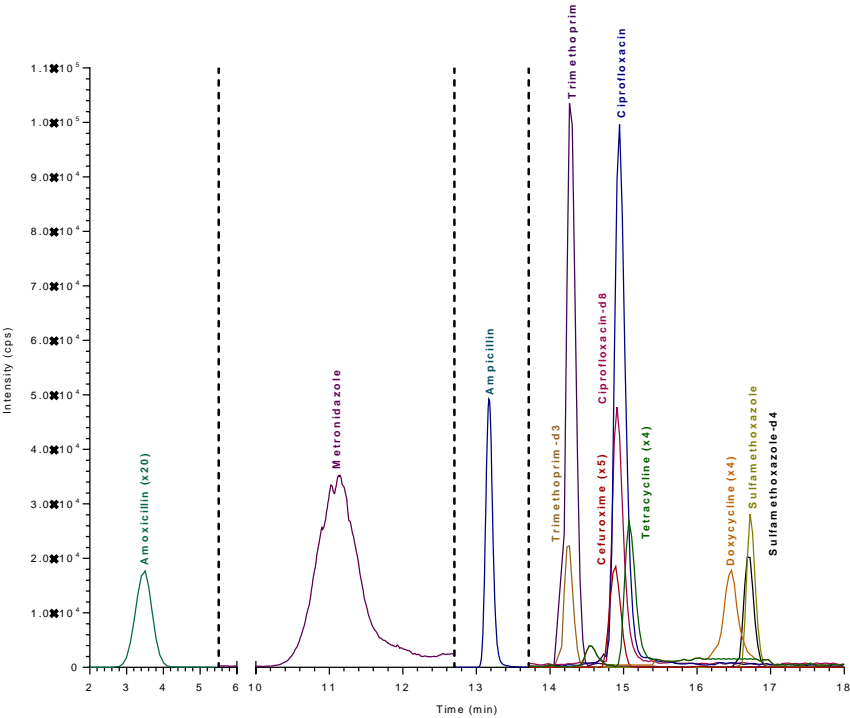

Supplement: Figure S1 — Chromatogram of a standard antibiotic-mix solution in a concentration of 5.0 ppm obtained from the final HPLC-MS/MS method. The segments are represented in dotted lines. (PDF) [file pone.0113055.s001.pdf]
