# Supplementary material for: Antibiotic Exposure in a Low-Income Country: Screening Urine Samples for Presence of Antibiotics and Antibiotic Resistance in Coagulase Negative Staphylococcal Contaminants
Source: PLoS One. 2014 Dec 2;9(12):e113055. doi: 10.1371/journal.pone.0113055 (PMC4251977; doi:10.1371/journal.pone.0113055)
Supplement: Table S1 — Compound specific settings in the final analytical LC-MS/MS method, applying ESI in positive and negative mode, together with the most intense precursor ions and product ions for the nine investigated compounds and the three internal standards. DP: Declustering potential, FP: Focusing potential, EP: Entrance potential, CE: Collision energy, CXP: Collision cell exit potential; V: Volt. Parameters changed from the in-house method are presented in bolt. (DOC) [file pone.0113055.s002.doc]

Supporting Information:

Table S1: Compound specific settings in the final analytical LC-MS/MS method, applying ESI in positive and negative mode, together with the most intense precursor ions and product ions for the nine investigated compounds and the three internal standards. DP: Declustering potential, FP: Focusing potential, EP: Entrance potential, CE: Collision energy, CXP: Collision cell exit potential; V: Volt. Parameters changed from the in-house method are presented in bolt.

| Compound | | Retention times (min) | Ionisations mode | Precursor ion (m/z) | Product ions quantify/qualify (*m/z*) | Dweel time (ms) | DP (V) | FP (V) | EP (V) | CE (V) | CXP (V) |
| --- | --- | --- | --- | --- | --- | --- | --- | --- | --- | --- | --- |
|  | |  |  |  |  |  |  |  |  |  |  |
| First period (0-5.5 min) | |  |  |  |  |  |  |  |  |  |  |
|  | Amoxicillin | 3.48 | Negative | 364.3 | 222.8/**302.8** | **3000** | -50 | -200 | -10 | -20 | -10 |
|  |  |  |  |  |  |  |  |  |  |  |  |
| Second period (5.5-12.7 min) | |  |  |  |  |  |  |  |  |  |  |
|  | Metronidazole | 11.1 | Positive | 172.1 | 128.0/**111.0** | **300** | 20 | 50 | 10 | 20 | 15 |
|  |  |  |  |  |  |  |  |  |  |  |  |
| Third period (12.7-13-7 min) | |  |  |  |  |  |  |  |  |  |  |
|  | Ampicillin | 13.2 | Negative | 348.1 | 206.8/**172.9** | **300** | -20 | -100 | -15 | -20 | -10 |
|  |  |  |  |  |  |  |  |  |  |  |  |
| Fourth period (13.7-30 min) | |  |  |  |  |  |  |  |  |  |  |
|  | Trimethoprim | 14.3 | Positive | 291.1 | 261.3/**275.2** | **50** | 30 | **100** | 10 | **35** | **6** |
|  | **D3-trimethoprim** | **14.3** | **Positive** | **294.1** | **264.3/275.2** | **50** | **30** | **100** | **10** | **35** | **6** |
|  | Ciprofloxacin | 14.9 | Positive | 332.0 | 314.2/**288.3** | **50** | **45** | 200 | 10 | **32** | **10** |
|  | **D8-ciprofloxacin** | **14.9** | **Positive** | **340.0** | **322.3/296.4** | **50** | **45** | **200** | **10** | **32** | **10** |
|  | Cefuroxime | 14.9 | Negative | 423.2 | 317.8/**206.7** | **50** | -20 | -100 | -10 | -10 | -15 |
|  | Tetracycline | 15.1 | Negative | 443.2 | 357.9/**166.9** | **50** | -20 | -200 | -10 | -30 | -10 |
|  | Doxycycline | 16.5 | Negative | 443.2 | 357.9/**166.9** | **50** | -20 | -200 | -10 | -30 | -10 |
|  | Sulfamethoxazole | 16.7 | Negative | 252.0 | 155.7 | **50** | -20 | -100 | -5 | -20 | -15 |
|  | **D4-sulfamethoxazole** | **16.7** | **Negative** | **256.0** | **159.7** | **50** | **-20** | **-100** | **-5** | **-20** | **-15** |
