# Supplementary material for: Antibiotic Exposure in a Low-Income Country: Screening Urine Samples for Presence of Antibiotics and Antibiotic Resistance in Coagulase Negative Staphylococcal Contaminants
Source: PLoS One. 2014 Dec 2;9(12):e113055. doi: 10.1371/journal.pone.0113055 (PMC4251977; doi:10.1371/journal.pone.0113055)
Supplement: Table S2 — Number of staphylococcal isolates and their antibiotic-resistance profiles to individual antimicrobial agents in isolates from KBTH in Accra and SODH in Dodowa. (DOC) [file pone.0113055.s003.doc]

Supporting Information:

Table S2: Number of staphylococcal isolates and their antibiotic-resistance profiles to individual antimicrobial agents in isolates from KBTH in Accra and SODH in Dodowa

|  | *S. haemolyticus* | | | | Non-*S. haemolyticus* | | | |
| --- | --- | --- | --- | --- | --- | --- | --- | --- |
|  | KBTH | | SODH | | KBTH | | SODH | |
|  | R | I + S | R | I + S | R | I + S | R | I + S |
|  |  |  |  |  |  |  |  |  |
| Penicillin V | 43 | 1 | 28 | 0 | 9 | 1 | 14 | 0 |
| Trimethoprim | 31 | 13 | 19 | 9 | 5 | 5 | 9 | 5 |
| Tetracycline | 29 | 15 | 20 | 8 | 2 | 8 | 10 | 4 |
| Chloramphenicol | 21 | 23 | 18 | 10 | 2 | 8 | 9 | 5 |
| Sulfonamide | 24 | 20 | 14 | 14 | 1 | 9 | 5 | 9 |
| Cefoxitin | 25 | 19 | 15 | 13 | 0 | 10 | 4 | 10 |
| Gentamicin | 22 | 22 | 15 | 13 | 1 | 9 | 2 | 12 |
| Rifampicin | 12 | 32 | 12 | 16 | 1 | 9 | 3 | 11 |
| Erythromycin | 12 | 32 | 4 | 24 | 2 | 8 | 1 | 13 |
| Clindamycin | 5 | 39 | 1 | 27 | 1 | 9 | 1 | 13 |
| Fusidic acid | 1 | 43 | 2 | 26 | 2 | 8 | 1 | 13 |
| Vancomycin | 0 | 44 | 0 | 28 | 0 | 10 | 0 | 14 |
|  |  |  |  |  |  |  |  |  |
| Total | 225 | 303 | 148 | 188 | 26 | 84 | 59 | 109 |
